# A preliminary report on oral glucose tolerance and antinociceptive activity tests conducted with methanol extract of *Xanthosoma violaceum* aerial parts

**DOI:** 10.1186/1472-6882-14-335

**Published:** 2014-09-13

**Authors:** Mohammad Faisal, Ahamed Ismail Hossain, Shahnaz Rahman, Rownak Jahan, Mohammed Rahmatullah

**Affiliations:** Department of Biotechnology and Genetic Engineering, University of Development Alternative, Dhanmondi Dhaka, 1209 Bangladesh; Department of Pharmacy, University of Development Alternative, Dhanmondi Dhaka, 1209 Bangladesh; Faculty of Life Sciences, University of Development Alternative, House No. 78, Road No. 11A (new), Dhanmondi Dhaka, 1209 Bangladesh

**Keywords:** Antihyperglycemic, *Xanthosoma violaceum*, OGTT, Antinociceptive, Araceae

## Abstract

**Background:**

*Xanthosoma violaceum* is commonly observed in fallow areas of Bangladesh but almost no scientific studies exist on this plant. Rural people consume the plant on a frequent basis. The objective of this study was to scientifically analyze the antinociceptive property of methanol extract of aerial parts of the plant along with antihyperglycemic activity.

**Methods:**

Antihyperglycemic activity was measured by oral glucose tolerance test (OGTT). Antinociceptive activity was determined by observed decreases in abdominal constrictions in intraperitoneally administered acetic acid-induced pain model in mice.

**Results:**

Administration of methanol extract of aerial parts led to dose-dependent and significant reductions in blood glucose levels in glucose-loaded mice. At doses of 50, 100, 200 and 400 mg per kg body weight, the extract reduced blood sugar levels by 19.3, 23.2, 31.8, and 47.1%, respectively compared to control animals. By comparison, a standard antihyperglycemic drug, glibenclamide, when administered at a dose of 10 mg per kg body weight, reduced blood glucose level by 48.9%. In antinociceptive activity tests, the extract at the above four doses reduced the number of abdominal constrictions by 41.4, 44.8, 48.3, and 55.2%, respectively. A standard pain relieving (antinociceptive) drug, aspirin, reduced the number of writhings by 31.0 and 51.7%, respectively, when administered at doses of 200 and 400 mg per kg body weight.

**Conclusion:**

To our knowledge, this is the first report on oral glucose tolerance and antinociceptive activity evaluation of aerial parts of the plant. Since the plant is widely available in Bangladesh, the aerial parts can be a readily available source for particularly the rural population for lowering blood sugar in diabetic patients and for alleviating pain.

## Background

*Xanthosoma violaceum* Schott (Araceae) is a common plant in Bangladesh and can be seen growing in fallow lands. In Bangladesh, it is known as ‘Dudh kochu’ and in English as “blue taro’. Aerial parts of the plant are consumed as vegetable by particularly the low income sections of the rural people, because it can be collected freely from the fallow lands. Until now, any scientific reports are practically non-existent on this plant. The only available report mentions that flavone-C-glycosides (apigenin 6-C-β-D-glucopyranosyl-8-C-β-D-apiofuranoside, as well as vitexin, isovitexin, isovitexin 4′-O-rhamnopyranoside, apigenin 6-C-[β-D-glucopyranosyl-(1-- > 6)-β-D-glucopyranoside], and apigenin 6,8-diC-β-D-glucopyranoside) with antioxidative properties are present in the leaves of the plant [1].

Antidiabetic, hypolipidemic and antioxidative effects have been noted with a related species of *X. violaceum*, namely, *Xanthosoma sagittifolium* corm extract [2]. Apigenin derivatives, vitexin and isovitexin have been reported in *X. violaceum* [1]. Apigenin has been reported to be able to regulate diabetes mellitus, as well as diabetes-induced thyroid dysfunction and lipid peroxidation in alloxan-induced diabetic mice [3]. Streptozotocin (STZ)-treated islets were observed to release more insulin in the presence of apigenin, isolated from *Teucrium polium*, than apigenin-non-treated islets [4]. Apigenin reportedly attenuated 2-deoxy-D-ribose-induced oxidative cell damage in HIT-T15 pancreatic β-cells [5]. Vitexin and isovitexin, isolated from leaves of *Ficus deltoidea*, has been reported to *in vivo* inhibit α-glucosidase activity [6].

Methanol extract of *Jatropha gossypifolia* aerial parts has been reported to exhibit analgesic and anti-inflammatory activity; apigenin, vitexin and isovitexin are known to be present in the leaves of this plant [7]. Methanol extract of aerial parts of *Ficus pumila* has also been reported to show analgesic and anti-inflammatory activities; apigenin was identified as one of the active ingredients [8].

Diabetes and pain are afflictions suffered by people throughout the world. Although medications are available, the rural people of Bangladesh often do not have access to or can afford these medications. As such, we had been screening various common Bangladeshi plants for their antihyperglycemic and antinociceptive properties [9–12]. Considering the presence of antidiabetic and analgesic components (apigenin, vitexin, isovitexin) present in *X. violaceum*, and considering that the corms of a related species *X. sagittifolium* have been reported to give antidiabetic effects, the objective of the present study was to conduct oral glucose tolerance test (OGTT) and acetic acid-induced gastric pain model test with methanol extract of aerial parts of *X. violaceum* towards evaluating the antihyperglycemic and antinociceptive potential of the extract.

## Methods

### Plant material collection

Aerial parts (leaves and stems) of *X. violaceum* were collected during September 2013 from Kawlar in Dhaka district, Bangladesh and taxonomically identified at the Bangladesh National Herbarium (Accession Number 38,592).

### Preparation of methanolic extract of aerial parts

Aerial parts were cut into small pieces, air-dried in the shade, and 100 g of dried and powdered leaves and stems was extracted with methanol (w:v ratio of 1:6, final weight of the extract 10.05 g). 100 g of dried and powdered leaves and stems were stirred continuously in 600 ml methanol for 70 minutes. The mixture was left overnight followed by fresh stirring for 70 minutes the following morning. The mixture was again left overnight followed by filtration. All procedures were conducted at ambient temperature (25°C). The filtrate was collected and evaporated at 40°C [9–12].

### Chemicals and drugs

Glibenclamide, aspirin, and glucose were obtained from Square Pharmaceuticals Ltd., Bangladesh. All other chemicals were of analytical grade.

### Animals

Swiss albino mice (male), which weighed between 14–19 g were used in the present study. The animals were obtained from International Centre for Diarrhoeal Disease Research, Bangladesh (ICDDR,B). The animals were acclimatized for three days prior to actual experiments. The study was conducted following approval by the Institutional Animal Ethical Committee of University of Development Alternative, Dhaka, Bangladesh (Approval No. 48/EC/2013/UODA).

### Oral glucose tolerance tests for evaluation of antihyperglycemic activity

Oral glucose tolerance tests were carried out as per the procedure previously described by Joy and Kuttan (1999) [13] with minor modifications. Briefly, fasted mice were grouped into six groups of five mice each. The various groups received different treatments like Group 1 received vehicle (1% Tween 80 in water, 10 ml/kg body weight) and served as control, Group 2 received standard drug (glibenclamide, 10 mg/kg body weight). Groups 3–6 received extract (MEXV) at doses of 50, 100, 200 and 400 mg per kg body weight. All substances were orally administered. Following a period of one hour, all mice were orally administered 2 g glucose/kg of body weight. Blood samples were collected 120 minutes after the glucose administration through puncturing heart. Blood glucose levels were measured by glucose oxidase method [14]. The percent lowering of blood glucose levels were calculated according to the formula described below.


where W_e_ and W_c_ represents the blood glucose concentration in glibenclamide or MEXV administered mice (Groups 2–6), and control mice (Group 1), respectively.

### Antinociceptive activity evaluation through abdominal writhing test

Antinociceptive activity of MEXV was examined as previously described [15]. Mice were divided into seven groups of five mice each. Group 1 served as control and was administered vehicle only. Groups 2 and 3 were orally administered the standard antinociceptive drug aspirin at doses of 200 and 400 mg per kg body weight, respectively. Groups 4–7 were administered MEXV at doses of 50, 100, 200 and 400 mg per kg body weight, respectively. Following a period of 60 minutes after oral administration of standard drug or MEXV, all mice were intraperitoneally injected with 1% acetic acid at a dose of 10 ml per kg body weight. A period of 5 minutes was given to each animal to ensure bioavailability and onset of chemically induced irritation of acetic acid [11], following which period, the number of abdominal constrictions (writhings) was counted for 10 min. The percent inhibitions of abdominal constrictions were calculated according to the formula given below.


where W_e_ and W_c_ represents the number of writhings in aspirin or MEXV administered mice (Groups 2–7), and control mice (Group 1), respectively.

### Acute toxicity test

Acute toxicity test was conducted as previously described [16]. Mice were divided into nine groups, each group consisting of six animals. Group 1 was given 1% Tween 80 in normal saline (2 ml per kg body weight). The other eight groups (Groups 2–9) were administered, respectively, 100, 200, 300, 600, 800, 1000, 2000 and 3000 mg of MEXV per kg body weight. All animals were closely observed for the next 8 hours to notice any behavioral changes or mortality and were kept under close observation for the next two weeks.

### Statistical analysis

Experimental values are expressed as mean ± SEM. Independent Sample *t*-test was carried out for statistical comparison. Statistical significance was considered to be indicated by a p value < 0.05 in all cases [12].

### Preliminary phytochemical screening

Preliminary phytochemical analysis of MEXV for presence of saponins, tannins, alkaloids, and flavonoids were conducted as described before [17].

## Results

### Preliminary screening of phytochemicals

Various tests conducted for presence of phytochemicals in MEXV indicated the presence of tannins, alkaloids, and flavonoids.

### Toxicity evaluation

The crude extract did not show any toxicity in mice even at the highest dose tested.

### Antihyperglycemic activity evaluation results

MEXV, when administered at doses of 50, 100, 200 and 400 mg per kg body weight, dose-dependently and significantly reduced the levels of blood glucose in mice. At these four doses, the percent lowering of blood glucose levels were, respectively, 19.3, 23.2, 31.8, and 47.1. By comparison, a standard antihyperglycemic drug, glibenclamide, when administered to mice at a dose of 10 mg per kg body weight, reduced blood glucose levels by 48.9%. The results are shown in Table 1 and indicate that the highest dose of MEXV was nearly equivalent to that of glibenclamide.

**Table 1:** **Effect of crude methanol extract of** ***X. violaceum*** **aerial parts (MEXV) on blood glucose level in hyperglycemic mice following 120 minutes of glucose loading**

Treatment	Dose (mg/kg body weight)	Blood glucose level (mmol/l)	% lowering of blood glucose level
Control	10 ml	5.60 ± 0.27	-
Glibenclamide	10 mg	2.86 ± 0.26	48.9*
(MEXV)	50 mg	4.52 ± 0.16	19.3*
(MEXV)	100 mg	4.30 ± 0.18	23.2*
(MEXV)	200 mg	3.82 ± 0.27	31.8*
(MEXV)	400 mg	2.96 ± 0.19	47.1*

All administrations were made orally. Values represented as mean ± SEM, (n = 5); *P < 0.05; significant compared to hyperglycemic control animals.

### Antinociceptive activity evaluation results

Dose-dependent and significant reductions in the number of abdominal constrictions induced by intraperitoneal administration of acetic acid were observed with MEXV. At doses of 50, 100, 200 and 400 mg per kg body weight, MEXV reduced the number of constrictions, respectively, by 41.4, 44.8, 48.3, and 55.2%. A standard antinociceptive drug, aspirin, when administered to experimental animals at doses of 200 and 400 mg per kg body weight, reduced the number of abdominal constrictions by 31.0 and 51.7%, respectively. Thus all the doses of MEXV exhibited greater antinociceptive activity than aspirin when administered at a dose of 200 mg per kg body weight. At the highest dose of 400 mg of MEXV tested, the extract showed better antinociceptive activity than even 400 mg per kg aspirin. The results are shown in Table 2 and suggest that the extract has powerful antinociceptive properties, more so than aspirin.

**Table 2:** **Antinociceptive effect of crude methanol extract of** ***X. violaceum*** **aerial parts (MEXV) in acetic acid-induced pain model mice**

Treatment	Dose (mg/kg body weight)	Mean number of abdominal constrictions	% inhibition
Control	10 ml	5.8 ± 0.37	-
Aspirin	200 mg	4.0 ± 0.84	31.0*
Aspirin	400 mg	2.8 ± 0.37	51.7*
(MEXV)	50 mg	3.4 ± 0.24	41.4*
(MEXV)	100 mg	3.2 ± 0.58	44.8*
(MEXV)	200 mg	3.0 ± 0.63	48.3*
(MEXV)	400 mg	2.6 ± 0.60	55.2*

All administrations (aspirin and extract) were made orally. Values represented as mean ± SEM, (n = 5); *P < 0.05; significant compared to control.

## Discussion

To our knowledge, this is the first reported instance of oral glucose tolerance and antinociceptive activity tests conducted with aerial parts of *X. violaceum*. The plant belongs to the Araceae family. Plant parts from other Araceae family plants have been previously reported for antihyperglycemic and antinociceptive activities, although the reports are few. Oligosaccharides isolated from *Amorphophallus konjac* have previously been shown to give hypoglycemic effect in streptozotocin (STZ)-induced diabetic model of isolated islets, and which has been hypothesized to be related with free radical attenuation and lower risks of islets damage from nitric oxide NO(*) radical [18]. The ameliorative potential of *Colocasia esculenta* tubers have been reported in STZ-induced diabetic nephropathy in rats [19]. Methanol extract of leaves of *C. esculenta* also reportedly demonstrated antihyperglycemic and antinociceptive potential [20].

Antinociceptive and antiinflammatory effect of *Anchomanes difformis* extract has been reported in rats against formalin-induced pain and egg albumin-induced inflammation [21]. The analgesic activity of methanol leaf extract of *Culcasia scandens* has been reported against acetic acid-induced writhings and formalin-induced paw licking in mice [22]. The analgesic activity of methanol extract of *Amorphophallus campanulatus* tubers has also been reported [23].

Any phytochemical constituent(s) responsible for the observed antihyperglycemic and antinociceptive effects were not isolated and identified in this preliminary study. However, phytochemical analysis of the crude extract showed presence of tannins, alkaloids and flavonoids. Also as discussed earlier, some reported constituents of the plant like apigenin, vitexin and isovitexin have reported antidiabetic and analgesic activities [1, 3–8]. Antidiabetic and antihyperlipidemic effects of ethanolic extract of whole plant of *Tridax procumbens* have been shown in STZ-induced diabetic rats. The crude extract was found to contain tannins, alkaloids, and flavonoids [24]. Hypoglycemic and tissue-protective effects have been seen with aqueous extract of *Persea americana* seeds in alloxan-induced diabetic rats [25]; the extract was also found to contain tannins, alkaloids, and flavonoids. Antinociceptive and antioxidant activities have been observed with ethanolic crude extract of leaves of *Ageratum conyzoides* from Bangladesh; phytochemical analysis of the crude extract revealed the presence of tannins, alkaloids, and flavonoids [26]. Thus these group of compounds, either singly or in combination may be responsible for the observed antihyperglycemic and antinociceptive effects in the present study.

## Conclusion

The results suggest that the extract merits further scientific attention for further isolation and identification of the bioactive component(s) responsible for the observed antihyperglycemic and antinociceptive effects.
